# GLP-1 (7–36) amide restores myocardial insulin sensitivity and prevents the progression of heart failure in senescent beagles

**DOI:** 10.1186/s12933-014-0115-x

**Published:** 2014-07-31

**Authors:** Melissa Chen, Franca S Angeli, You-tang Shen, Richard P Shannon

**Affiliations:** Departments of Medicine, University of Pennsylvania Perelman School of Medicine, Philadelphia, PA USA; Department of Medicine, University of Virginia School of Medicine, Philadelphia, PA USA; University of Virginia Health System, PO Box 800813, 22908 Charlottesville, VA USA

**Keywords:** Glucagon-like pepetide-1, Dilated cardiomyopathy, Myocardial insulin resistance, Aging

## Abstract

**Background:**

We previously demonstrated that older beagles have impaired whole body and myocardial insulin responsiveness (MIR), and that glucagon-like peptide-1 (GLP-1 [7–36] amide) improves MIR in young beagles with dilated cardiomyopathy (DCM). Here, we sought to determine if aging alone predisposes to an accelerated course of DCM, and if GLP-1 [7–36] amide would restore MIR and impact the course of DCM in older beagles.

**Methods:**

Eight young beagles (Young-Control) and sixteen old beagles underwent chronic left ventricle (LV) instrumentation. Seven old beagles were treated with GLP-1 (7–36) amide (2.5 pmol/kg/min) for 2 weeks prior to instrumentation and for 35 days thereafter (Old + GLP-1), while other 9 served as control (Old-Control). All dogs underwent baseline metabolic determinations and LV biopsy for mitochondria isolation prior to the development of DCM induced by rapid pacing (240 min^−1^). Hemodynamic measurements were performed routinely as heart failure progressed.

**Results:**

At baseline, all old beagles had elevated non-esterifed fatty acids (NEFA), and impaired MIR. GLP-1 reduced plasma NEFA (Old-Control: 853 ± 34; Old + GLP-1: 531 ± 33 μmol/L, p < 0.02), improved MIR (Old-Control: 289 ± 54; Old + GLP-1: 512 ± 44 mg/min/100 mg, p < 0.05), and increased uncoupling protein-3 (UCP-3) expression in isolated mitochondria. Compared to the Young-Control, the Old-Controls experienced an accelerated course of DCM (7 days versus 29 days, p < 0.005) and excess mortality, while the Old + GLP-1 experienced increased latency to the onset of DCM (7 days versus 23 days, p < 0.005) and reduced mortality.

**Conclusion:**

Aging is associated with myocardial insulin resistance, which predispose to an accelerated course of DCM. GLP-1 treatment is associated with increased MIR and protection against an accelerated course of DCM in older beagles.

## Introduction

The aging population in the western world continues to be burdened by a disproportionate share of cardiovascular (CV) disease and the morbidity and mortality of cardiovascular conditions such as heart failure is increased among the elderly [1,2]. Cardiovascular risk factors such as hypertension and diabetes accrue in older populations contributing to the increased burden of CV disease. However, it remains uncertain as to how much of the burden is a consequence of these recognized risk factors and how much is attributable to aging alone [3].

Prior studies from our laboratory have demonstrated that aging in beagles is associated with the development of both whole body and myocardial insulin resistance, independent of obesity or inactivity [4]. Other studies have demonstrated similar findings in rodents with a greater emphasis on skeletal muscle insulin resistance than on myocardial insulin resistance [5]. However, myocardial insulin resistance may be of clinical importance, given the preference for stressed heart to increase glucose utilization and reduce non-esterifed fatty acids (NEFA) oxidation [6]. Our laboratory has determined that the cellular basis for myocardial insulin resistance in senescent beagles is associated with mitochondrial abnormalities leading to accumulation of unoxidized NEFA in cardiac myocytes [4]. The mitochondrial abnormalities include decreased expression of key regulators of oxidative phosphorylation including mitochondrial cytochrome oxidase-1 (MCO) and uncoupling protein-3 (UCP-3) [6]. Others have shown that senescent cardiac mitochondria have increased generation of reactive oxygen species leading to peroxidation of key mitochondrial proteins [7,8] predisposing to mitochondrial swelling and disruption. Moreover, in the setting of advanced age, mitochondrial biogenesis, myocyte energetics and cell survival may be compromised [9-11], contributing to an increased risk of heart failure in this population [12].

Therefore, new strategies designed to improve mitochondrial function and restore myocardial insulin resistance in older patients may prove cardio-protective. One such candidate is glucagon-like peptide −1 (GLP-1 [7-36] amide), an incretin hormone that has shown to have potential cardiovascular benefits beyond glycemic control [13-18]. We have previous demonstrated that GLP-1 [7-36] amide was associated with increased insulin action and improved mitochondrial function in young dogs with pacing induced cardiomyopathy [19]. Accordingly, the purpose of the present study was to determine if aging alone predisposes to an accelerated course of dilated cardiomyopathy (DCM) induced by rapid pacing in the absence of conventional cardiovascular risks. A second goal was to determine if myocardial insulin responsiveness could be restored in the senescent myocardium through treatment with GLP-1 (7–36) amide. A third goal was to determine whether preservation of mitochondrial proteins and restoration of myocardial insulin responsiveness would protect the older beagles from an accelerated course of DCM when exposed to a similar pacing stress.

## Methods

**Figure 1:**
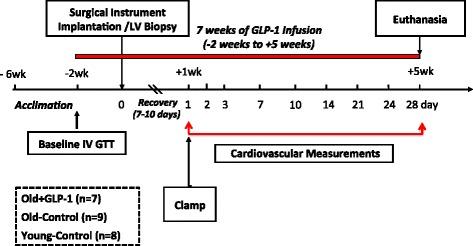
**Schematic illustration of the study design and time points for assessment.** LV, left ventricle; IV GTT, intravenous glucose tolerance test; Clamp, hyperinsulinemic-euglycemic clamp.

Eight young (Young-Control; age 3 years) and 16 old (10–12 years) beagles, weighing 14–18 kg were used in the present study. Figure 1 summarizes the study design. Animal age was confirmed through dental examinations by the veterinary staff. All dogs were carefully screened for all disease known to occur commonly in canines and studied in accordance with the “Guide for the Care and Use of Laboratory Animal Resources” [DHHS Publication No (NIH) 86–23, Revised 1996] and the guidelines of the Institutional Animal Care and Use Committees of the University of Pennsylvania.

### Acclimation and surgical instrumentation

The animals were acclimated to the research laboratory for 6 weeks prior to surgical instrumentation. They were fed a standard chow once daily with a fixed carbohydrate and fat content, and underwent a supervised exercise regimen on a treadmill each day for 30 minutes four times per week for 6 weeks prior to surgical instrumentation as previously described [4].

All dogs underwent sterile surgical instrumentation as previously described [15,20,21]. The dogs were allowed to recover from the surgical procedure for 7 days, during which time they were treated with analgesics and antibiotics and trained to rest quietly on the experimental table in a conscious, unrestrained state.

### GLP-1 (7–36) amide intervention protocol

After 4 weeks of training, the older dogs were randomized to receive either GLP-1 (7–36) amide though a continuous intravenous infusion at a dose of 2.5 pmol/kg/min (Old + GLP-1; n = 7) or saline (3 ml/day) control (Old-Control; n = 9) using a MiniMed infusion system. The GLP-1 (7–36) amide treatment was initiated 2 weeks prior to surgery and was continued through surgery, recovery (1 week) and during the rapid pacing stimulus for 28 days (Figure 1). Synthetic GLP-1 was mixed in 2.8 ml of normal saline and 0.2 ml of fresh plasma prepared from each animal as previously described [15]. A 99% pure, pyrogen-free and sterile, single lot of GLP-1 (7–36) amide synthesized in the protein/peptide core facility of the Massachusetts General Hospital was used in this study. Net peptide content was used for all calculations.

### Metabolic determinations

All metabolic parameters were measured at 8 am, following an overnight fast. After 4 weeks of training, all dogs underwent baseline blood sampling and an intravenous glucose challenge (250 mg/kg IV with 5–10 minute sampling of glucose and insulin for 60 minutes) to determine the magnitude of whole body insulin resistance between young and old beagles prior to exposure to GLP-1. In addition, plasma norepinephrine, insulin, glucagon, adiponectin, non-esterified fatty acids (NEFA), and glucose were measured as previously described [20].

Additionally, whole body and myocardial insulin sensitivity was assessed using the hyperinsulinemic-euglycemic clamp after surgical instrumentation and prior to the pacing insult to assess the effects of 3 weeks of GLP-1 treatment [15,19]. Briefly, in the fasting state, a primed, constant infusion of insulin (480 pmol • m^−2^ • min^−1^) was administered for 120 minutes to create a steady state concentration of plasma insulin (~1000 pmol/L). Arterial glucose concentrations were measured every 5 minutes and glucose was infused to maintain plasma glucose concentrations at 5 mmol/L ±5%. Myocardial glucose and NEFA balance and coronary blood flow were sampled every 15 minutes to determine myocardial glucose uptake. Trans-myocardial substrate balance was calculated as the difference between arterial and coronary sinus content. Basal myocardial substrate uptake was calculated as the product of myocardial substrate balance and coronary blood flow (CBF) [15,19].

### Induction of dilated cardiomyopathy and hemodynamic measurements

After baseline metabolic and hemodynamic measurements, all animals underwent rapid right ventricular pacing using a customized pacemaker at 240 beats/ minute. The rapid pacing was confirmed daily. Animals underwent hemodynamic evaluation at baseline (prior to pacing), daily for the first 3 days after pacing and then every 3 days for the next 28 days or until pre-specified hemodynamic parameters indicative of advanced DCM were reached. Advanced DCM was defined by the presence of depressed LV contractility (LVdP/dt) <1500 mmHg/sec, increased LV end-diastolic pressure (LVEDP) > 30 mmHg, reduced cardiac output (CO) < 1.8 L/min, and increased LV end-diastolic dimensions (LVEDD) >45 mm. In addition, stroke volume (SV), and myocardial oxygen consumption (MVO_2_) (product of the left circumflex CBF and the myocardial arterio-venous O_2_ content difference) were calculated. Coronary flow reserve (maximal coronary blood flow in response to adenosine- resting CBF, ml/min) was determined before and after the development of DCM.

### Mitochondrial isolation

LV myocardial biopsy for isolation of intrafibrillar mitochondria (IFM) was performed at baseline during surgical instrumentation. This timing allowed for comparison of mitochondrial proteins between Young and Old-Control and Old + GLP-1 treated beagles. Crude mitochondrial isolates were prepared from canine myocardium using a trypsin digestion procedure as described previously [4,15]. Briefly, after confirming the purity of the mitochondrial preparations by the absence of Na+/K + ATPase activity, isolated mitochondrial samples were re-suspended to a final protein concentration of 0.5 μg/mL in a buffer at a final concentration of 125 mM Tris–HCl, pH 6.8, 20% (v/v) glycerol, 5% (v/v) β-mercaptoethanol, and 0.1% (w/v) bromphenol blue. Membrane samples for UCP-3, SDHA, and MCO were subjected to electrophoretic separation by SDS-PAGE [4,22]. Proteins resolved were transferred onto PVDF membrane (Immobilon™-P^SQ^, Millipore Corp., Bedford, MA) at a constant voltage (100 V) [23]. Nonspecific membrane protein binding sites were blocked [5% (w/v) dry milk in Tris-buffered saline with 0.1% (v/v) Tween-20 (TTBS)] and then membranes were probed with polyclonal rabbit anti-UCP-3 antibody (1:10,000) (Alpha Diagnostics, San Antonio, TX), or monoclonal mouse anti-MCO antibody (1:100,000) (Abcam Inc., Cambridge, MA). The immunoreactive proteins were detected by use of an enhanced horseradish peroxidase/luminol chemiluminescence reaction kit (Perkin Elmer Life Sciences, Boston, MA) and exposed to X-ray film (Hyperfilm ECL™, Amersham Pharmacia Biotech, Piscataway, NJ). Densitometric analysis of the bands was carried out using a Personal Densitometer SI and ImageQuant™ Software (Molecular Dynamics, Sunnyvale, CA).

Mitochondrial lipid peroxidation was assessed by levels of malondialdehyde (MDA), a marker of lipid oxidation using lipid peroxidation assay kit (Calbiochem, San Diego, CA) as previously described [24]. To prevent sample oxidation, extracts were normalized to 1–1.5 mg/ml in re-suspension buffer with 5 mM butylated hydroxyl toluene. For each reaction, a 200-μl sample/standard was added to 650-μl chromogenic reagent and 150 μl of 12 N HCl. After reaction at 45°C for 60 min, the samples were cooled at 4°C and centrifuged at 10,000 *g* for 5 min. The supernatants were collected, and absorbance at 586 nm was recorded. MDA concentration was calculated using a standard curve. All measurements were performed in duplicate.

### Statistical analysis

Data are expressed as the mean value ± SEM. Differences in hemodynamic and metabolic responses between the groups were determined by two-way ANOVA**.** Where differences were detected over time, a post hoc Student’s Newman Keuls’s test was performed to determine differences at respective time points. Differences in mitochondrial proteins and metabolic parameters were determined using Student T-test for unpaired data. A Bonferroni correction was used when multiple comparisons were evaluated. A level of p < 0.05 was considered statistically significant.

## Results

### The effects of age on baseline body mass index and metabolic parameters

Table 1 illustrates the effects of age on body mass index and metabolic parameters after 4 weeks of training and prior to GLP-1 infusion and surgical instrumentation. At baseline, there was no difference in body weight, body mass index or abdominal girth between groups. Fasting plasma glucose levels were normal while plasma insulin levels (p < 0.01) and NEFA levels (p < 0.001) were significantly higher in the Old-Control and Old + GLP-1 groups suggesting insulin resistance. There were no differences in plasma adiponectin or leptin levels. Notably, plasma arterial norepinephrine was also increased (p < 0.001) in the Old-Control and Old + GLP-1 groups.

**Table 1:** **Effects of age on baseline body mass index and metabolic parameters**

	***Young***	***Old***				
	**Control**	**Old-Control**		**Old + GLP-1**		
	**n = 8**	**n = 9**	p value*	**n = 7**	p value*	p value **
Age (years)	3.5 ± 1	11.2 ± 1	<0.01	11.9 ± 1	<0.01	NS
BMI (kg/w^2^)	25 ± 0.6	26 ± 0.7	NS	27 ± 1	NS	NS
Body Weight (kg)	15 ± 3	15 ± 3	NS	16 ± 2	NS	NS
Glucose (mmol/L)	5.2 ± 0.2	5.0 ± 0.2	NS	4.9 ± 0.3	NS	NS
NEFA (μmol/L)	363 ± 52	897 ± 67	<0.01	913 ± 75	<0.01	NS
Insulin (pmol/L)	33 ± 9	110 ± 13	<0.01	99 ± 20	<0.01	NS
Glucagon (pq/ml)	26 ± 2	32 ± 9	NS	38 ± 8	NS	NS
Adiponectin (μg/ml)	2.9 ± 0.6	2.7 ± 0.9	NS	2.9 ± 0.5	NS	NS
NE (pmol/ml)	56 ± 13	396 ± 52	<0.01	422 ± 40	<0.01	NS

Data is presented as Mean ± SEM; *Compared to young; **Old-Control compared to Old + GLP-1; Old-Control, old beagles placebo; Old + GLP-1, old beagles GLP-1 treated; BMI, body mass index; NEFA, Non-esterified fatty acids; NE, plasma arterial norepinephrine.

**Figure 2:**
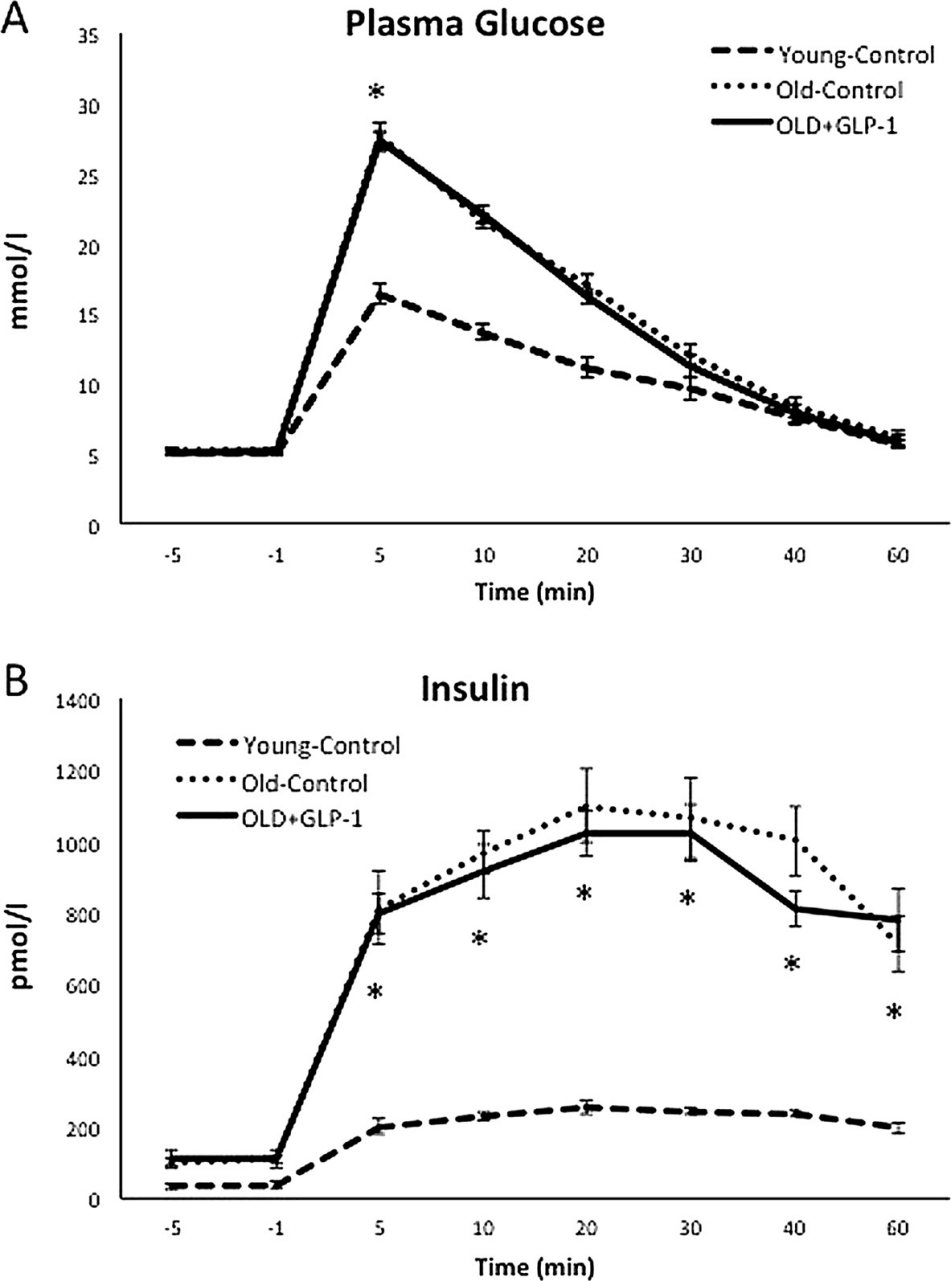
**Baseline intravenous glucose challenge 4 weeks after training.** Young-Control (n = 8), Old-Control (n = 9), and Old + GLP-1 (n = 7) dogs underwent to an intravenous glucose challenge prior to randomization to GLP-1 infusion. **(A)** Higher peak of plasma glucose response and **(B)** greater increase in plasma insulin in the older dogs, both suggesting whole body insulin resistance in the older groups compared to Young-Control animals. IV GTT, intravenous glucose tolerance test; *p < 0.01.

The intravenous glucose challenge in young and older beagles at baseline prior to GLP-1 infusion was associated with a higher peak plasma glucose response in the Old-Control and Old + GLP-1 groups (Figure 2A) and a markedly greater increase in plasma insulin (Figure 2B), suggesting whole body insulin resistance in the Old-Control and Old + GLP-1 groups compared to Young-Control. Notably, there was no difference in the degree of whole body insulin resistance or any other baseline parameter between the Old-Control and Old + GLP-1 groups prior to GLP-1 infusion (Table 1).

### Age and the effects of GLP-1Infusion on hemodynamic parameters prior to onset of pacing induced DCM

After 3 weeks of GLP-1 treatment and 7 days from surgical instrumentation (Figure 1), LV and systemic hemodynamics were assessed in all groups (Table 2). There were no significant differences in the LV systolic or end diastolic pressures, LV dP/dt, or LV dimensions between groups. Old-Control beagles had impaired LV isovolumic relaxation time constants (tau: 25 ± 2 vs. 18 ± 1 msec, p < 0.001), higher mean arterial pressures (107 ± 7 vs. 89 ± 3 mmHg, p < 0.05) and systemic vascular resistance (3421 ± 224 vs 2649 ± 267 dyne-sec-cm^−5^, p < 0.05) compared to Young-Control, but comparable heart rate, cardiac output, and LV ejection fraction. Old-Control dogs had higher resting MVO_2_ compared to Young-Control (313 ± 39 vs 150 ± 21 mlO_2_/min, p < 0.01). GLP-1 treatment improved isovolumic relaxation in Old + GLP-1 compared to Old-Control (p < 0.05). GLP-1 had no significant effects on resting cardiac output or coronary blood flow. Mean arterial pressure, systemic vascular resistance and MVO_2_ tended to be lower in Old + GLP-1 dogs after 3 weeks of treatment compared to Old-Control, although the differences did not reach statistical significance.

**Table 2:** **Aging and GLP-1 on left ventricular and systemic hemodynamics after 3 weeks of GLP-1 treatment and before the onset of DCM**

	***Young***	***Old***			
	**Control**	**Old-Control**		**Old + GLP-1**	
	**n = 8**	**n = 9**	p value*	**n = 7**	p value*
LVESP ( mmHg)	118 ± 4	132 ± 10	NS	130 ± 10	NS
LVEDP (mmHg)	9 ± 1	11 ± 2	NS	10 ± 3	NS
LV dP/dt ( mmHg /sec)	2881 ± 121	2801 ± 176	NS	2912 ± 200	NS
LV tau (msec)	18 ± 1	25 ± 2	<0.01	19 ± 1†	NS
MAP ( mmHg)	89 ± 3	107 ± 7	<0.05	102 ± 5	<0.05
Heart Rate (min-1 )	90 ± 4	101 ± 10	NS	99 ± 12	NS
CO (L/min)	2.7 ± 0.3	2.5 ± 0.3	NS	2.5 ± 0.4	NS
LVEF (%)	54 ± 4	50 ± 5	NS	51 ± 4	NS
SVR (dyne-sec-cm-5)	2649 ± 267	3421 ± 224	<0.05	3164 ± 123	NS
MVO_2_ (ml O_2_/min)	150 ± 21	313 ± 39	<0.01	243 ± 22†	<0.01
Coronary Flow (ml/min)	25 ± 3	26 ± 4	NS	27 ± 4	NS
LVEDD (mm)	40 ± 3	40 ± 3	NS	41 ± 3	NS
LVESD (mm)	33 ± 2	34 ± 3	NS	33 ± 3	NS

Data is presented as Mean ± SEM; *Compared to young; † p < 0.05 compared to Old-Control; Old-Control, old dogs placebo; Old + GLP-1, old dogs GLP-1 treated; LVESP, left ventricle end-systolic pressure; LVEDP, left ventricle end-diastolic pressure; LV dP/dt, left ventricle rate of rise of left ventricular pressure; tau, isovolumic relaxation time constants; MAP, mean arterial pressure; CO, cardiac output; LVEF, left ventricle ejection fraction; SVR, systemic vascular resistance; MVO_2,_ myocardial oxygen consumption, LVEDD, left ventricle end-diastolic diameter; LVESD, left ventricle end-systolic diameters.

### Age predisposes progression of pacing induced DCM

**Figure 3:**
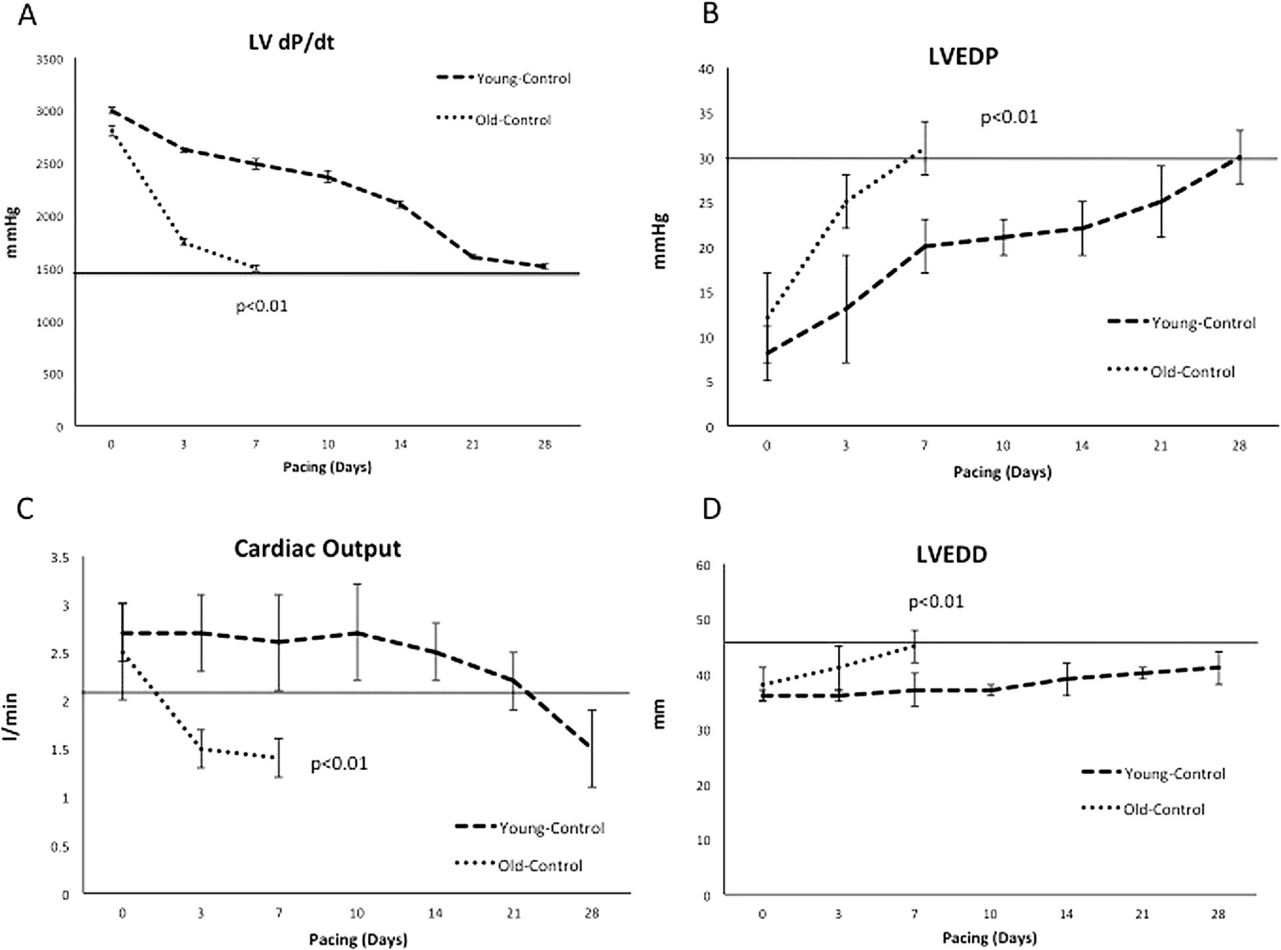
**The effects of aging infusion on the time course to the development of DCM.** Despite comparable baseline hemodynamics, Old-Control had a remarkably accelerated course of pacing induced DCM compared to Young-Control as demonstrated by: **(A)** early drop of LV dP/dt and **(B)** cardiac output, **(C)** rapid rise of LVEDP, and **(D)** early enlargement of left ventricle. LV dP/dt, left ventricle rate of rise of left ventricular pressure; LVEDP, left ventricle end-diastolic pressure; LVEDD, left ventricle end-diastolic diameter.

Figure 3 illustrates the time course of the development of hemodynamically significant dilated cardiomyopathy following the initiation of rapid pacing. LV dP/dt decreased to less than 1,500 mmHg/sec within 7 days in Old-Control dogs compared to 25 days in Young-Control (p < 0.001, Figure 3A). Cardiac output declined to less than 1.7 l/min within 3 days in Old-Control compared to 28 days in Young-Control (p < 0.001, Figure 3B), while LV end diastolic pressure rose to greater than 30 mmHg within 7 days in Old-Control compared to 28 days in Young-Control (p < 0.001, Figure 3C). Finally, LV dilatation occurred within 3 days in Old-Control compared to 28 days in Young-Control (p < 0.001, Figure 3D). Notably, 33% (n = 3) of the Old-Control dogs died before reaching the pre-specified hemodynamic end point compared to 0% mortality in the Young-Control at 28 days. Thus, despite comparable baseline hemodynamics, Old-Control dogs had a remarkably accelerated course of pacing induced DCM.

### GLP-1 infusion restores myocardial substrate uptake in older dogs

**Figure 4:**
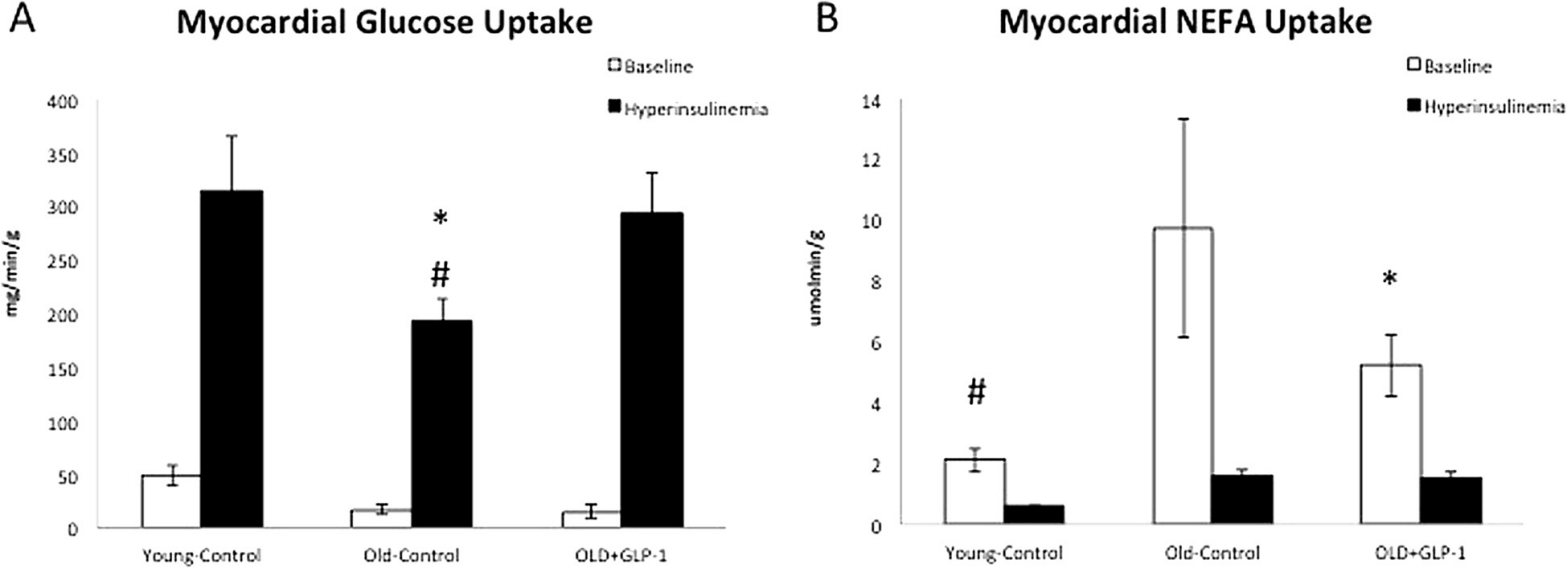
**Effects of age and GLP-1 infusion on myocardial glucose and non-esterified fatty acids uptake.** GLP-1 treatment improved basal and insulin stimulated myocardial glucose uptake **(A)** and suppressed basal NEFA uptake compared to Old-Control dogs **(B)**. NEFA, non-esterified fatty acids; *Old-Control versus Old + GLP-1, p < 0.05; # Old-Control versus Young-Control #p < 0.01.

The beneficial effects of GLP-1 infusion on myocardial metabolism were assessed under conditions of the hyperinsulinemic-euglycemic clamp. Both basal and insulin stimulated myocardial glucose uptake were reduced in Old-Control dogs (Figure 4A) while basal myocardial NEFA uptake was increased compared to Young-Control (Figure 4B). Hyperinsulinemia suppressed myocardial NEFA uptake and to a greater extent in Old-Control compared to Young-Control (Figure 4B). In contrast, GLP-1 infusion improved basal and insulin stimulated myocardial glucose uptake in Old + GLP-1 and suppressed basal NEFA uptake compared to Old-Control dogs (Figure 4A).

**Figure 5:**
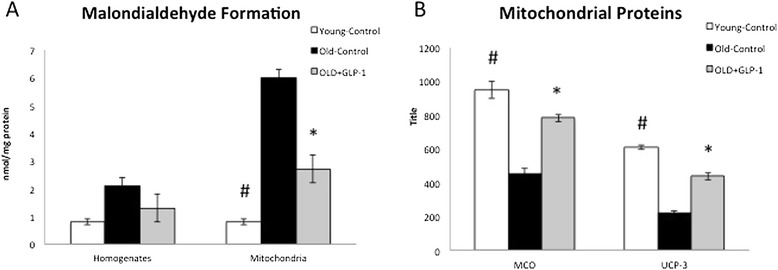
**Effects of age and GLP-1 infusion on reactive oxygen species and mitochondrial protein expression.** Aging was associated with higher levels of malondialdehyde **(A)** and decreased mitochondrial expression of UCP-3 and MCO **(B)** compared to Young-Control. GLP-1 treatment decreased malondialdehyde abundance **(A)** and increased mitochondrial protein expression **(B)** in Old + GLP-1 compared to Old-Control. MCO, mitochondrial cytochrome oxidase-1; UCP-3, uncoupling protein-3. *Old-Control versus Old + GLP-1, p < 0.05; #Old-Control versus Young-Control #p < 0.01.

Moreover, GLP-1 infusion had a positive impact on reactive oxygen species and mitochondrial protein expression in Old + GLP-1. Aging was associated with increased cellular and mitochondrial reactive oxygen species (ROS) compared to Young-Control as demonstrated by higher levels of MDA, a marker of lipid peroxidation, in the Old-Control dogs (Figure 5A). Aging was also associated with decreased mitochondrial expression of UCP-3 and MCO in cardiomyocytes compared to Young-Control (Figure 5B). Interestingly, GLP-1 infusion increased mitochondrial protein expression and decreased MDA abundance compared to Old-Control suggesting that GLP-1 may ameliorate underlying mitochondrial protein abnormalities and reduce ROS observed in the cardiomyocytes with aging.

### GLP-1 infusion preserves coronary flow reserve

**Figure 6:**
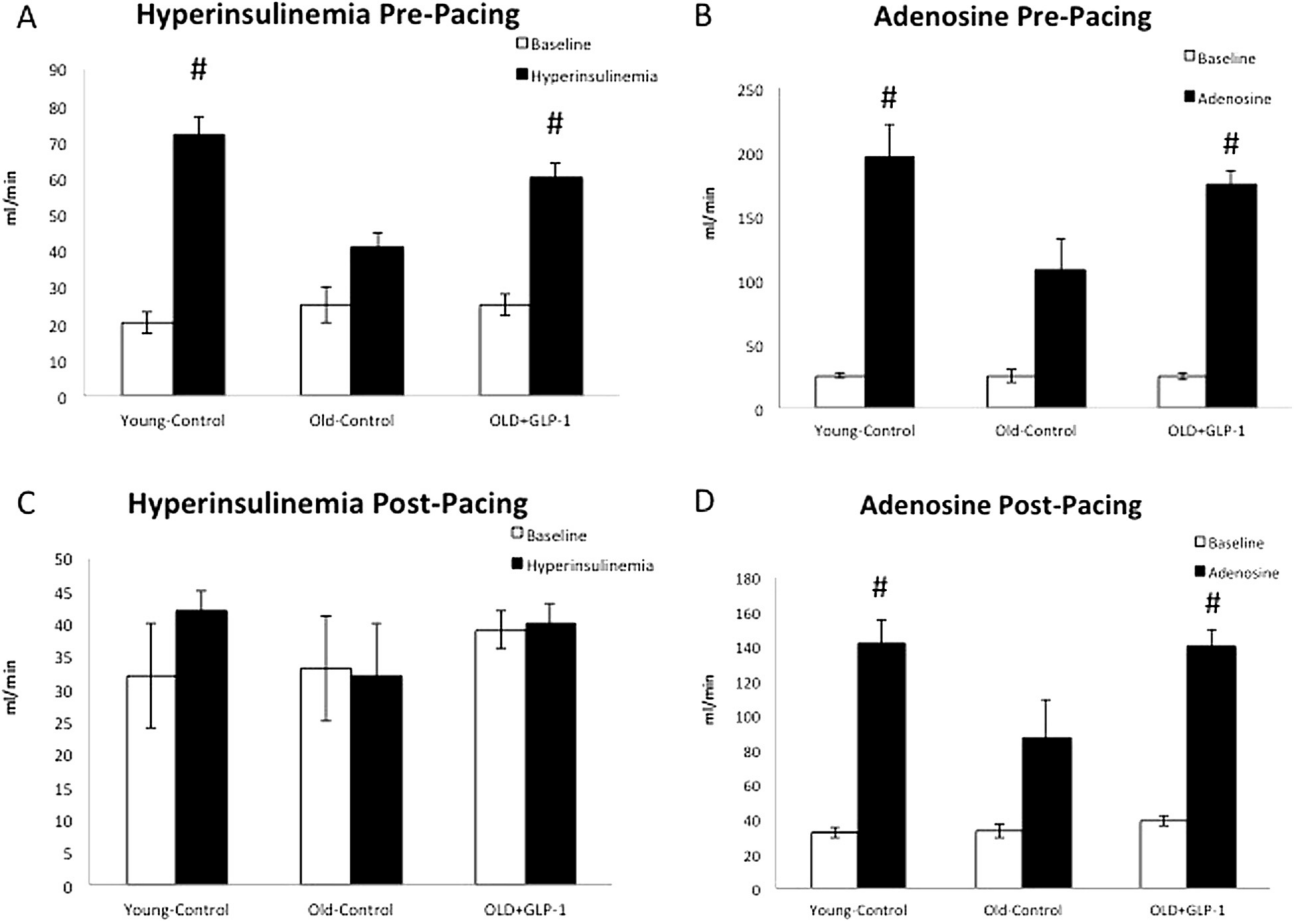
**Basal and provoked coronary blood flow responses before and after pacing.** Basal and provoked coronary blood flow in response to hyperinsulinemia pre-pacing **(A)** and after onset of dilated cardiommyopathy **(B)**. Basal and provoked coronary blood flow in response to and near maximal coronary vasodilator doses of adenosine pre pacing **(C)** and at the onset of dilated cardiomyopathy **(D)**. # p < 0.01 compared to baseline and to Old-Control.

There were no differences in basal coronary blood flow responses between groups either before (Figure 6A) or after the development of DCM (Figure 6B). However, the vasodilator response to hyperinsulinemia was markedly attenuated in Old-Control dogs at baseline and abolished completely following the development of DCM (Figure 6B). Notably, Old + GLP-1 dogs demonstrated preserved coronary vasodilator response to hyperinsulinemia in the basal state, but not following the development of advanced DCM (Figure 6A and B). Similarly, the coronary blood flow response to adenosine was reduced in Old-Control dogs both under basal conditions and following DCM (Figure 6C and D). Interestingly, Old + GLP-1 dogs showed preserved flow reserve under basal conditions and better preservation of flow reserve following the development of DCM compared to Old-Control (Figure 6C and D).

### GLP-1 protects old beagles from an accelerated course of DCM

**Figure 7:**
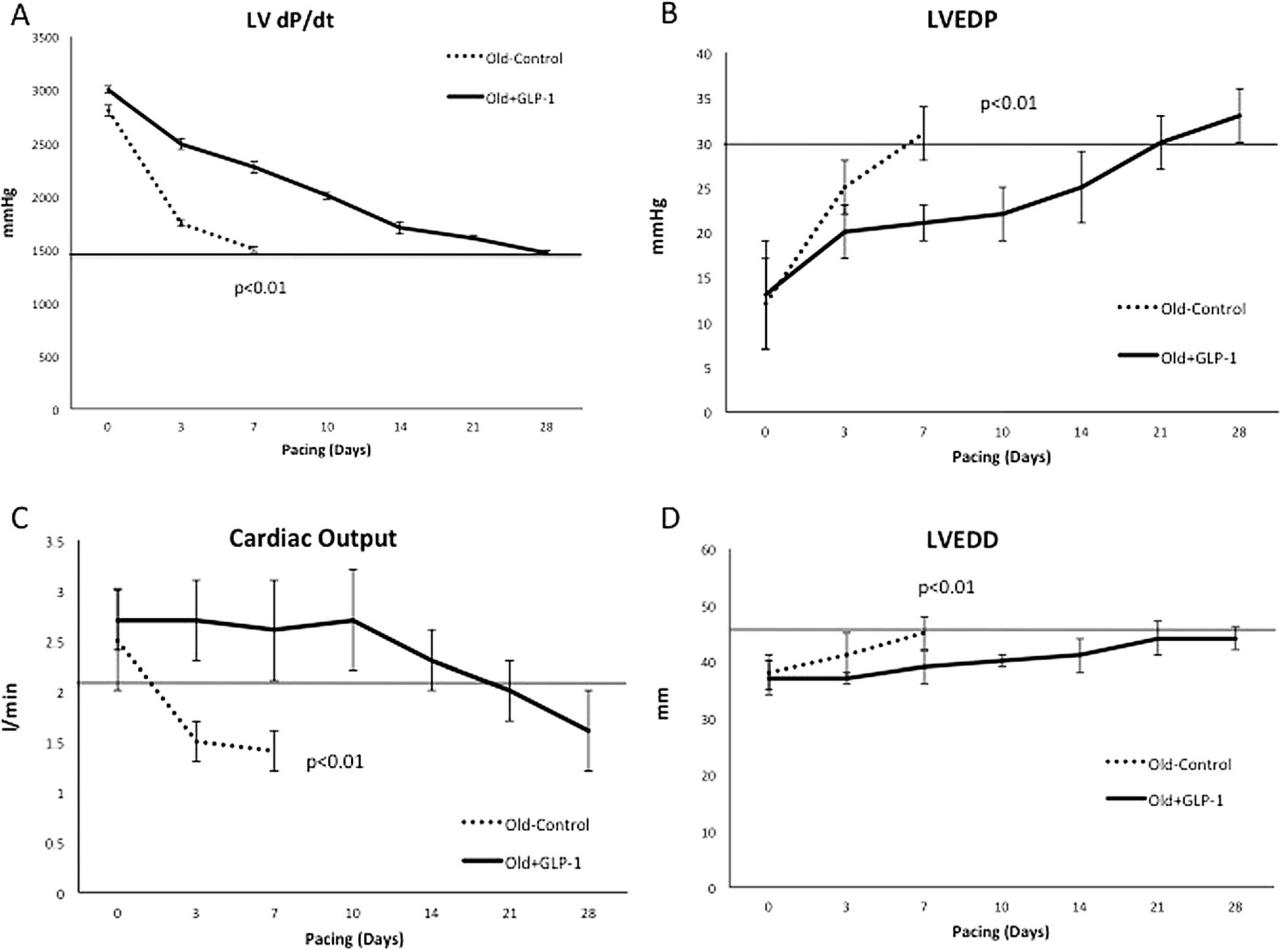
**The Effects of GLP-1 infusion on the time course to the development of DCM.** Despite comparable baseline hemodynamics, Old-Control had a remarkably accelerated course of pacing induced DCM compared to Old + GLP-1 as demonstrated by: **(A)** early drop of LV dP/dt and **(B)** cardiac output, **(C)** rapid rise of LVEDP, and **(D)** early enlargement of left ventricle. LV dP/dt, left ventricle rate of rise of left ventricular pressure; LVEDP, left ventricle end-diastolic pressure; LVEDD, left ventricle end-diastolic diameter.

GLP-1 infusion and the associated improvement in myocardial insulin action, mitochondrial protein expression and coronary flow reserve was associated with a prolonged course of dilated cardiomyopathy and increased survival in Old + GLP-1 compared to Old-Control (Figure 7). LV dP/dt decreased to less than 1,500 mmHg/sec within 7 days in Old-Control dogs with myocardial insulin resistance compared to 24 days in Old + GLP-1 dogs (p < 0.01, Figure 7A). Cardiac output declined to less than 1.7 l/min within 3 days in Old-Control compared to 28 days in Old + GLP-1 dogs (p < 0.01, Figure 7B), while LV end diastolic pressure rose to greater than 30 mmHg within 7 days in Old-Control compared to 21 days in Old + GLP-1 dogs (p < 0.01, Figure 7C). Finally, LV dilatation occurred within 3 days in Old-Control compared to 28 days in Old + GLP-1 dogs (p < 0.001, Figure 7D). Interestingly, Old + GLP-1 dogs survived to 28 days similar to that seen in Young-Control, but in contrast to the premature mortality observed in Old-Control. Compare to Young-Control **(**Figure 3) Old + GLP-1 dogs did not differ statistically in any of the hemodynamic parameters during the progression of pacing induced DCM.

## Discussion

In the present study, we observed that old dogs with myocardial insulin resistance developed an accelerated course of dilated cardiomyopathy in response to rapid ventricular pacing compared to Young-Control. Treatment with a continuous infusion of GLP-1(7–36) amide improved myocardial insulin sensitivity, reduced myocardial mitochondrial oxidative stress, preserved expression of key mitochondrial proteins associated with oxidative balance (UCP-3) and mitochondrial respiration (MCO), and preserved coronary flow reserve culminating in an improved clinical course and reduced mortality following rapid pacing. These findings underscore the role of mitochondrial dysfunction and associated impaired myocardial insulin responsiveness in the progression of heart failure in advanced age.

Aging has been associated with altered mitochondrial protein expression and reduced energetic reserve in studies of skeletal muscle [25,26]. These alterations have been linked to the cellular mechanisms of insulin resistance, mediated through increased accumulation of NEFA [4], features which have been associated with increased reactive oxygen species production in senescent mitochondria [27]. Under conditions in which ROS production exceeds anti-oxidative capacity, increased UCP-3 activity dissipates the electromotive force and reduces mitochondrial ROS production [28]. A consequence of the decreased transmenbrane potential (∆φ) is reduced electromotive force for ATP generation, which may become relevant under circumstances of increased energetic demand, as occurs with pacing induced heart failure [15,29]. When these physiological adaptive responses are further compromised by alterations in components of the electron transfer chain, such as MCO or decreased expression or function of UCP-3, further ROS generation leads to lipid peroxidation and eventually mitochondrial permeability transition, and ultimately necrotic cell death [7,22].

In the present study we observed myocardial insulin resistance and altered mitochondrial ROS generation in association with decreased MCO, and UCP-3 expression in intrafibrillar myocardial mitochondria in older beagles. We focused on IFM as the most relevant component given their role in myocardial contractility and because age-associated changes seem to affect IFM preferentially [30]. Decreased UCP-3 expression would be expected to further impair the mitochondrial capacity to mitigate ROS leading to lipid peroxidation and further mitochondrial dysfunction [31].

We have shown previously that GLP-1 infusion improves cardiovascular function when administered for 48 hours to young dogs with severe dilated cardiomyopathy [19]. We have also shown that the hemodynamic improvement was associated with both increased myocardial glucose uptake and increased expression of key components of the mitochondrial electron transport chain (ETC.), specifically MCO, the expression of which was significantly reduced in dilated cardiomyopathy. Furthermore, GLP-1 treatment was associated with reduced mitochondrial ROS production and increased UCP-3 expression. UCP-3 has been shown to be an important buffer against excess free radical accumulation within the mitochondrial matrix (29). We have also shown that normal aging in older beagles is associated with similar changes in the absence of heart failure (4). Here, we observe a similar effect of GLP-1 infusion on UCP-3 and MCO and also observe better preservation of these components following rapid pacing in old animals without baseline heart failure. Together, these salutary changes in mitochondrial protein expression were associated with slowing the progression of heart failure in the GLP-1 treated animals. Thus, both aging and heart failure appear to be associated with impaired mitochondrial function and myocardial glucose utilization and the effects appear to be synergistic.

We have also previously shown that the development of advanced heart failure in conscious dogs is associated with impaired coronary flow and vasodilator reserve in response to coronary vasodilators [20]. Here we demonstrate that aging alone in the presence of myocardial insulin resistance is associated with impaired coronary flow reserve in response to sub-maximal doses of adenosine. Importantly, these data are the first to confirm that GLP-1 treatment improves coronary flow responses to insulin and adenosine. Furthermore, coronary flow reserve was better preserved in advanced heart failure in the Old + GLP-1 compared to Old-Control. This is the first demonstration of a vascular protective effect of GLP-1 on coronary flow reserve in a model of non-ischemic DCM.

There is an extensive literature that has demonstrated the salutary effects of both DPP-4 inhibitors [32] and DPP-4 resistant long acting receptor analogs [33] in mitigating ischemia- reperfusion injury and reducing infarct size in rodent models through a PKA dependent mechanism. These findings underscore the importance of GLP-1 receptor medicated signaling in ischemia-reperfusion syndromes. Our findings are the first to document significant salutary effects of GLP-1 (7–36) amide in a large animal model of aging and heart failure. The findings are notable in light of the recent clinical trials data that have suggested that DPP-4 inhibitors increase the risk of heart failure hospitalizations in patients with Type 2 diabetes [34]. A critical difference is the fact that in our study, the native peptide is metabolized to the active metabolite, GLP-1 (9–36), which we [35] have shown to be biologically active, particularly vasoactive. These additive effects of the metabolite are not present with DPP-4 inhibitors or DPP-4 resistant GLP-1 receptor agonists. Secondly, the continuous infusion of the native peptide resulted in steady state pharmacological concentrations of total GLP-1 (~100 pM) while DPP-4 inhibitors result in physiological levels (20 pM). These differences may not only be relevant to our findings in older beagles but also explain why clinical studies of heart failure patients [16] and post-operative patients [36] have shown benefits with continuous infusions of the native peptide GLP-1 (7–36) amide.

### Limitations

The findings of increased mitochondrial ROS, reduced mitochondrial protein expression, and impaired myocardial insulin resistance were associations that do not prove causality in terms of the accelerated course of DCM in Old-Control. Other cellular process may also be deranged which could contribute to the response. Nonetheless, GLP-1 treatment mitigated the mitochondrial and metabolic abnormalities and protected Old-GLP-1against an accelerated course. Secondly, we measured mitochondrial protein expression not function. Additional studies detailing mitochondria abundance and morphology may help to further understand the role of mitochondrial dysfunction in this model and the extent to which GLP-1 may influence mitochondrial biogenesis and autophagy. Thirdly, it took several years to complete this study as the availability of senescent beagles was rate limiting. We began using MDA as a method to assess lipid peroxidation and continued to do so for the purposes of consistency over time. We recognize that currently there may be better quantitative methods of lipid peroxidation quantification*.* Finally, GLP-1 treatment was associated with improved coronary flow responses, suggesting vascular protective effects of GLP-1. While we cannot ascertain the relative contribution of these favorable changes to the improved clinical outcome in Old + GLP-1 dogs, these data demonstrate that GLP-1 treatment has multiple salutary cardiovascular effects in heart failure beyond glycemic control.

## Conclusions

Aging and associated mitochondrial dysfunction and insulin resistance predispose to an accelerated course of dilated cardiomyopathy and early mortality. Infusion of GLP-1 (7–36) amide is associated with reduced myocardial mitochondrial ROS and improved myocardial insulin sensitivity and protection against an accelerated course of dilated cardiomyopathy and early mortality in older beagles, suggesting salutary cardiovascular effects in heart failure beyond glycemic control.

## Abbreviations

BMI: Body mass index
Clamp: Hyperinsulinemic-eyglycemic clamp
CO: Cardiac output
CV: Cardiovascular
DCM: Dilated cardiomyopathy
GLP: Glucagon like peptide
IFM: Intrafibrillar mitochondria
IV GTT: Intravenous glucose tolerance test
LV dP/dt: Left ventricle rate of rise of left ventricular pressure
LV: Left ventricle
LVEDD: Left ventricle end-diastolic diameter
LVEDD: Left ventricle end-diastolic diameter
LVEDP: Left ventricle end-diastolic pressure
LVEF: Left ventricle ejection fraction
LVESD: Left ventricle end-systolic diameters.
MAP: Mean arterial pressure
MCO: Mitochondrial cytochrome oxidase-1
MIR: Myocardial insulin responsiveness
MVO_2_: Myocardial oxygen consumption
NE: Plasma arterial norepinephrine
NEFA: Non-esterified fatty acids
SVR: Systemic vascular resistance
tau: Isovolumic relaxation time constants
UCP-3: Uncoupling protein-3
